# Effects of TiO_2_ nanoparticles on wheat (*Triticum aestivum L*.) seedlings cultivated under super-elevated and normal CO_2_ conditions

**DOI:** 10.1371/journal.pone.0178088

**Published:** 2017-05-30

**Authors:** Fuping Jiang, Yunze Shen, Chuanxin Ma, Xiaowen Zhang, Weidong Cao, Yukui Rui

**Affiliations:** 1 College of Resources and Environmental Sciences, China Agricultural University, Beijing, China; 2 China Astronaut Research and Training Center, Beijing, China; 3 Stockbridge School of Agriculture, University of Massachusetts, Amherst, Massachusetts, United States of America; 4 Department of Analytical Chemistry, The Connecticut Agricultural Experiment Station, New Haven, Connecticut, United States of America; 5 Key Laboratory of Plant Nutrition and Fertilizer, Ministry of Agriculture of the P.R. China/ Institute of Agricultural Resources and Regional Planning, Chinese Academy of Agricultural Sciences, Beijing, China; 6 Qinghai Academy of Agricultural and Forestry, Qinghai University, Xining, China; MEXICO

## Abstract

Concerns over the potential risks of nanomaterials to ecosystem have been raised, as it is highly possible that nanomaterials could be released to the environment and result in adverse effects on living organisms. Carbon dioxide (CO_2_) is one of the main greenhouse gases. The level of CO_2_ keeps increasing and subsequently causes a series of environmental problems, especially for agricultural crops. In the present study, we investigated the effects of TiO_2_ NPs on wheat seedlings cultivated under super-elevated CO_2_ conditions (5000 mg/L CO_2_) and under normal CO_2_ conditions (400 mg/L CO_2_). Compared to the normal CO_2_ condition, wheat grown under the elevated CO_2_ condition showed increases of root biomass and large numbers of lateral roots. Under both CO_2_ cultivation conditions, the abscisic acid (ABA) content in wheat seedlings increased with increasing concentrations of TiO_2_ NPs. The indolepropioponic acid (IPA) and jasmonic acid (JA) content notably decreased in plants grown under super-elevated CO_2_ conditions, while the JA content increased with increasing concentrations of TiO_2_ NPs. Ti accumulation showed a dose-response manner in both wheat shoots and roots as TiO_2_ NPs concentrations increased. Additionally, the presence of elevated CO_2_ significantly promoted Ti accumulation and translocation in wheat treated with certain concentrations of TiO_2_ NPs. This study will be of benefit to the understanding of the joint effects and physiological mechanism of high-CO_2_ and nanoparticle to terrestrial plants.

## Introduction

Nanotechnology is one of the revolutionary fields in science and technology and it is expected to contribute to advances in sustainability, including energy generation, conservation, storage, and conversion [1]. Nanoparticles (NPs), which are defined as particles in which at least one of the dimensions does not exceed 100 nm, are being applied in diverse industries including cosmetics, medicine, food and food packaging, bioremediation, and paints and coatings [1–4]. Sales of nanomaterial products are expected to reach 3 trillion dollars by 2020 [1]. With the increasing use and types of nano-products, the risks of NPs are receiving considerable attention. However, there is still a lack of information about interactions at the molecular level between NPs and biological systems [5–7]. At present, the NPs most commonly released into the environment include carbonaceous nanoparticles, metal oxides, quantum dots, zero-valent metals, and nanopolymers [8].

Higher plants are a major component of the food chain, and play an important role in ecosystem. Therefore, studying the toxic effects of NPs on plants will help us to understand the uptake, transportation, transformation, and degradation of NPs in the environment. Although the uptake, transport, and toxicity of NPs into plants are still not fully understood, it is thought that all of these factors are affected by the composition, size, and shape of NPs [8,9]. Previous studies have shown that NPs can enter the vascular system of plants and be transported to other plant tissues, and movements over short distances are favored [10]. Kurepa et al. [11] demonstrated that nanoconjugates could traverse cell walls to enter plant cells, and accumulate in specific subcellular locations.

To date, NPs have been demonstrated to have positive, negative, or no effects on plants, and the effect depends on the type of NPs and the plant species [12–15]. Nanoscale Zero Valent Iron (nZVI) was shown to inhibit seed germination and shoot growth of ryegrass, barley, and flax both in aqueous suspensions and soil [16]. In another study, FeO NPs inhibited plant growth by adversely affecting arbuscular mycorrhizal fungi [17]. Nanoparticles of ZnO, Fe_3_O_4_, and SiO_2_ were shown to have toxic effects on *Arabidopsis thaliana*, while Al_2_O_3_ NPs did not [18]. Recently, Fe_2_O_3_ NPs with superb adsorption capacity were successfully used as fertilizer to replace traditional Fe fertilizer [19].

Poorly soluble TiO_2_ NPs are used widely in paints, plastics, cosmetics, and catalysts [20,21]. Interactions between TiO_2_ NPs and plants have raised concern, and several studies have explored the effects of these NPs on plants [21–27]. Compared with bulk TiO_2_, nano-anatase TiO_2_ resulted in significant increases in biomass, total nitrogen, oxygen, chlorophyll, and protein contents of spinach leaves [28]. Also, nano-anatase TiO_2_ promoted spectral responses, which led to increased primary electron separation, electron transfer, and light energy conversion of the D1/D2/Cyt b559 complex by binding to this complex [29]. However, TiO_2_ NPs have also been shown to negatively affect plants. For example, at higher concentrations, TiO_2_ NPs were shown to delay germination, reduce the mitotic index, and inhibit root elongation of *Vicia narbonensis* L. and *Zea may*s L. [30].

Concerns over the potential risks of nanomaterials to ecosystem have been raised, as it is highly possible that nanomaterials could be released to the environment and result in adverse effects to living organisms. Carbon dioxide (CO_2_) is one of the main greenhouse gases. The level of CO_2_ keeps increasing and subsequently causes a series of environmental problems, especially for agricultural crops[31,32]. In the present study, we investigated the effects of TiO_2_ NPs on wheat seedlings cultivated under super-elevated CO_2_ conditions (5000 mg/L CO_2_) and under normal CO_2_ conditions (400 mg/L CO_2_). Representative parameters such as biomass, root length, phytohormone were determined to understand plant’s defense and response to abiotic stress caused by TiO_2_ NPs. Additionally, TiO_2_ NPs uptake was studied using ICP-MS.

## Materials and methods

### Characterization of TiO_2_ NPs

The TiO_2_ NPs (purity, ≥99.5%) were purchased from Sigma-Aldrich Inc. (3050 Spruce Street, Saint Louis, MO 63103, USA). These NPs were anatase and in a fine white powder form. The size and morphology of TiO_2_ NPs were determined by transmission electronic microscopy (TEM, JEM-200, Japan). Wheat seeds (Zhongmai 11) were purchased from the Chinese Academy of Agricultural Sciences. All of the chemicals were of analytical grade and were purchased from Signofarm Chemical Research Co., Ltd (Shanghai, China).

### Hydroponic culture

Wheat seeds were germinated after surface sterilization and were immersed in deionized water. The seeds were first incubated in a growth chamber (GZP-250B, Hengyu, China) at 24°C in the dark for 48 h. Then, the germinated seedlings were cultivated in the same growth chamber at 24°C under a 12-h light (light intensity of 15000 Lux)/12-h dark photoperiod for an additional 48 h. After germination, ten uniform seedlings were selected and transferred into plastic tubes containing 50 mL Hoagland’s solution [composition (mmol/L): Ca(NO_3_)_2_·4H_2_O, 2; KH_2_PO_4_, 0.1; MgSO_4_·7H_2_O, 0.5; 0.1 mM KCl, 0.1; 0.7 mM K_2_SO_4_, 0.7; 10 μM H_3_BO_3_, 10×10^−3^; MnSO_4_·H_2_O, 0.5×10^−3^; ZnSO_4_·7H_2_O,1×10^−3^; CuSO_4_·5H_2_O, 0.2×10^−3^; (NH_4_)_6_Mo_7_O_24_·4H_2_O, 0.01×10^−3^; 100 μM Fe-EDTA, 100×10^−3^]. The hydroponic assay was conducted using the CELSS (controlled ecological life support system) Integration Experiment Platform (CIEP) [33,34] in plant growth chamber in January, 2016. Healthy wheat seedlings were selected for TiO_2_ exposure in the CELSS under controlled conditions [25°C, 55% relative humidity, 12-h light (light intensity of 15000 Lux)/12-h dark photoperiod]. The CELSS was supplied with treated air with atmospheric pressure and 5000 mg/L CO_2_, and the plant growth chamber was supplied with fresh air in which the concentration of CO_2_ was 400 mg/L. The concentration of CO_2_ in the CELSS was more than 11 folds higher than in the plant growth chamber and all the other components in the air were the same. Under the two different CO_2_ concentrations, four TiO_2_ NPs treatment groups were applied: 10 mg/L, 100 mg/L, and 1000 mg/L, and a control without TiO_2_ NPs. There were three replicates in each treatment under 400 mg/L CO_2_ concentrations and four replicates under 5000 mg/L CO_2_ concentrations. TiO_2_ NPs were dispersed in Hoagland’s solution by sonication for 30 min. During the 14-day exposure, deionized water was added every day to compensate for the evaporation losses. The TiO_2_ NPs amended Hoagland’s solution was replaced completely at day 5 and day 10.

### Biomass measurement

At harvest, the treated seedlings were firstly washed with tap water for five times and then thoroughly washed with deionized water to remove impurities adsorbed on the surface of tissues. For each treatment, four seedlings were selected randomly to measure number of lateral root, root length and shoot height. And three of seedlings were selected to determined fresh weight of roots and shoots, separately.

### Quantification of Ti content by inductively coupled plasma mass spectroscopy

Dried shoots were ground to a fine powder, and then digested with a mixture of concentrated plasma-pure HNO_3_ and H_2_O_2_ (v/v, 6:1) in microwave digestion system (Ultra WAVE, Milestone, Italy). The obtained residual solutions were then diluted with deionized water and analyzed using inductively coupled plasma mass spectroscopy (ICP-MS).

### Phytohormone determination

According to previous studies [7,35], the concentrations of indole acetic acid (IAA), gibberellins (GA_s_), abscisic acid (ABA), jasmonic acid (JA), brassinosteroid (BR), zeatin riboside (ZR), dihydrozeatin riboside (DHZR), and indolepropioponic acid (IPA) were determined by ELISA methods.

### Data analysis

All results are presented as mean±standard deviation (SD). Data were analyzed using SPSS 20.0 (SPSS Inc., Chicago, IL, USA). Statistical analysis was performed using one-way analysis of variance (ANOVA) followed by LSD test and independent samples t-test. A confidence of 95% (P<0.05) was considered significant in all cases.

## Results and discussion

### Characterization of TiO_2_ NPs

**Fig 1:**
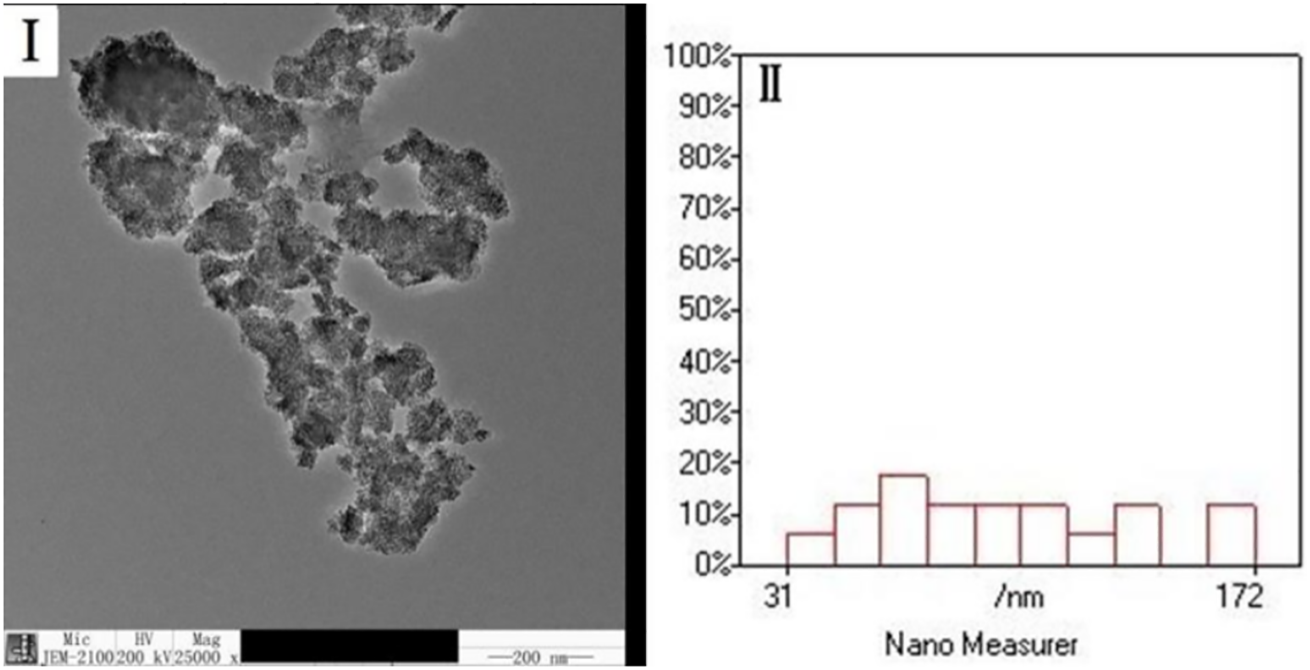
TEM image and particle size distribution of TiO_2_ NPs.

The morphology of NPs is presented in Fig 1 and S1 Fig. The NPs were easily agglomerated. The NPs were not uniform and had a wide size distribution with diameter ranging from 32 nm to 171 nm. Such aggregation was also evident in previous studies [3,20,23].

### Growth of wheat seedlings

**Fig 2:**
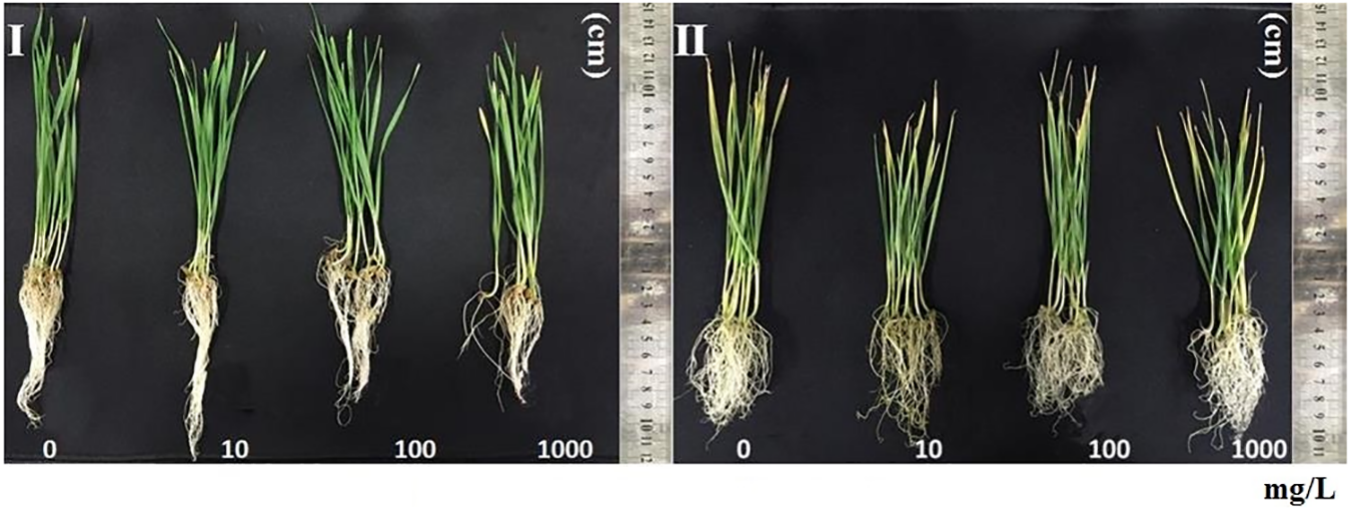
Phenotypic images of wheat seedlings in different concentrations of TiO2 NPs treatments with or without super elevated CO_2_. (I) Seedlings grown in different concentrations of TiO_2_ NPs under normal CO_2_ conditions in a plant growth chamber. (II) Seedlings grown in different concentrations of TiO_2_ NPs under super-elevated CO_2_ conditions.

As shown in Fig 2, the seedlings grown under super-elevated CO_2_ turned to yellow or light brown, whereas those in normal CO_2_ conditions were green and healthy. This result indicated that the elevated CO_2_ adversely notably impacted on the wheat seedlings.

### Effects of TiO_2_ NPs on seedling biomass, root elongation, and shoot height

**Fig 3:**
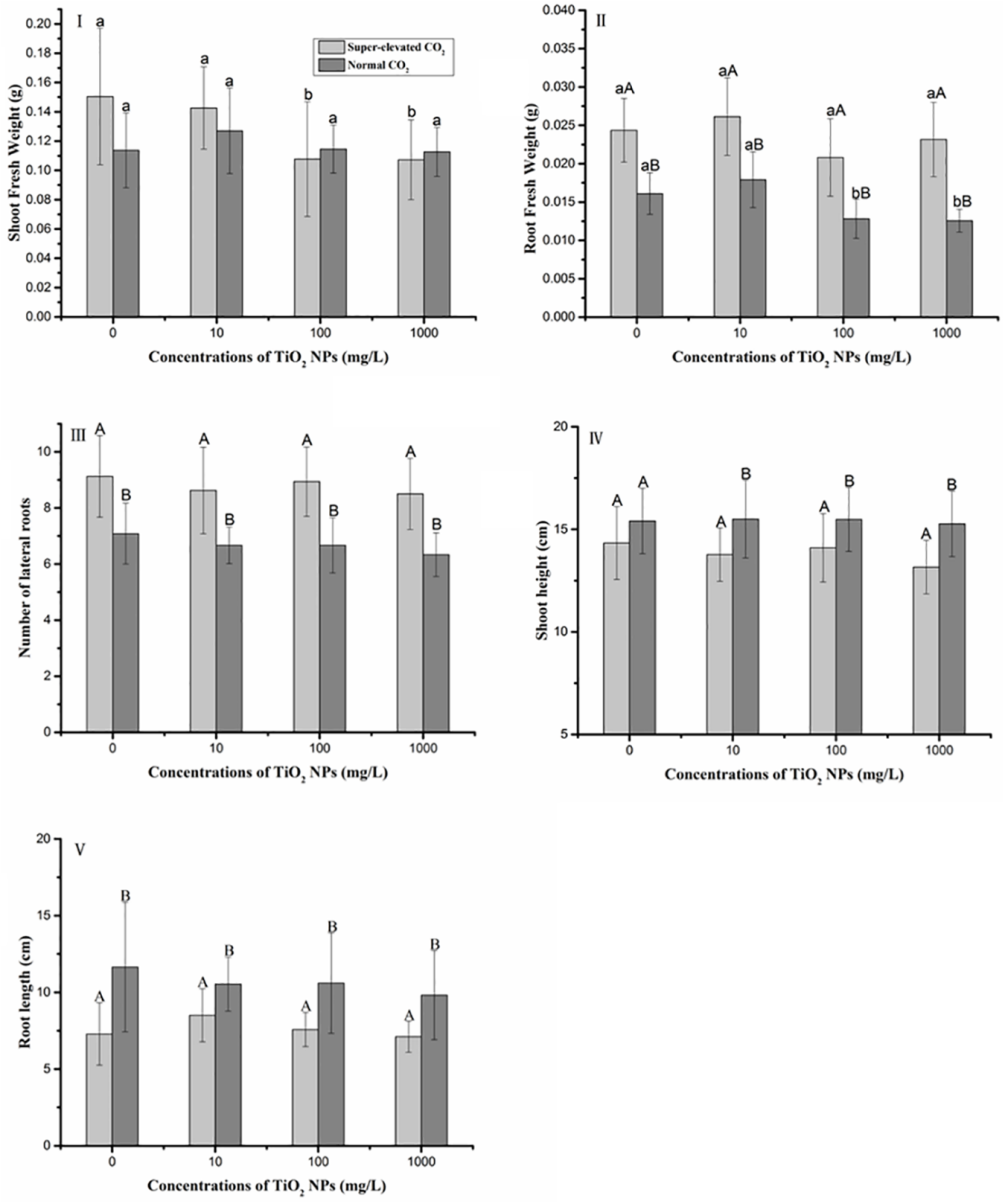
Effects of TiO_2_ NPs on seedling biomass and number of lateral roots. Values are mean±SD, error bars represent standard deviation (sample size, n = 12 for I and II, n = 16 for III, IV and V). Lower letters represent significant difference at p<0.05 among TiO_2_ NPs treatments under the same CO_2_ conditions; Upper letters represent significant difference at p<0.05 between super-elevated CO_2_ and normal CO_2_ conditions at the same TiO_2_ NPs concentration.

As shown in Fig 3I, shoot biomass decreased slightly with the concentration of TiO_2_ NPs increasing in seedlings grown under super-elevated CO_2_ conditions, while no significant difference of shoot biomass in all three TiO_2_ NPs treatments was found under normal CO_2_ conditions. Compared with super-elevated CO_2_, shoot height (Fig 3IV) was a little higher under normal CO_2_ condition. Upon exposure to the same concentration of TiO_2_ NPs, super-elevated CO_2_ did not significantly alter the shoot biomass (Fig 3I) or shoot height (Fig 3IV) and compared with those treated with normal CO_2_.

Root biomass was higher under super-elevated CO_2_ conditions than under normal CO_2_ conditions (Fig 3II). The results aligned with a previous study which demonstrated that elevated CO_2_ could stimulate root growth[36]. Additionally, compared with control groups, root fresh weight significantly decreased in 100 and 1000 mg/L TiO_2_ NPs under normal CO_2_ conditions, while no similar trends were observed among different TiO_2_ NPs concentrations under super-elevated CO_2_ conditions.

Plants grown under elevated CO_2_ produced more lateral roots than those grown under normal CO_2_ conditions. In the super-elevated CO_2_ and normal CO_2_ treatments, there was no significant difference in lateral root abundance among all three TiO_2_ NPs concentrations (Fig 3III). As shown in Fig 3V, similar trend was evident in root biomass. However, super-elevated CO_2_ significantly affected root length. According to the results, parameters except shoot fresh weight under super-elevated CO_2_ conditions and root fresh weight under normal CO_2_ conditions, there was no significant difference among CK, 10, 100 and 1000 mg/L TiO_2_ NPs treatments under same CO_2_ circumstance. Several previous studies also agreed with the findings that TiO_2_ NPs exhibited no toxic effects on plants regardless of exposure concentrations. [3,24,37] Additionally, it can’t be excluded that the sample sizes might be too low to detect to actual effects.

**Fig 4:**
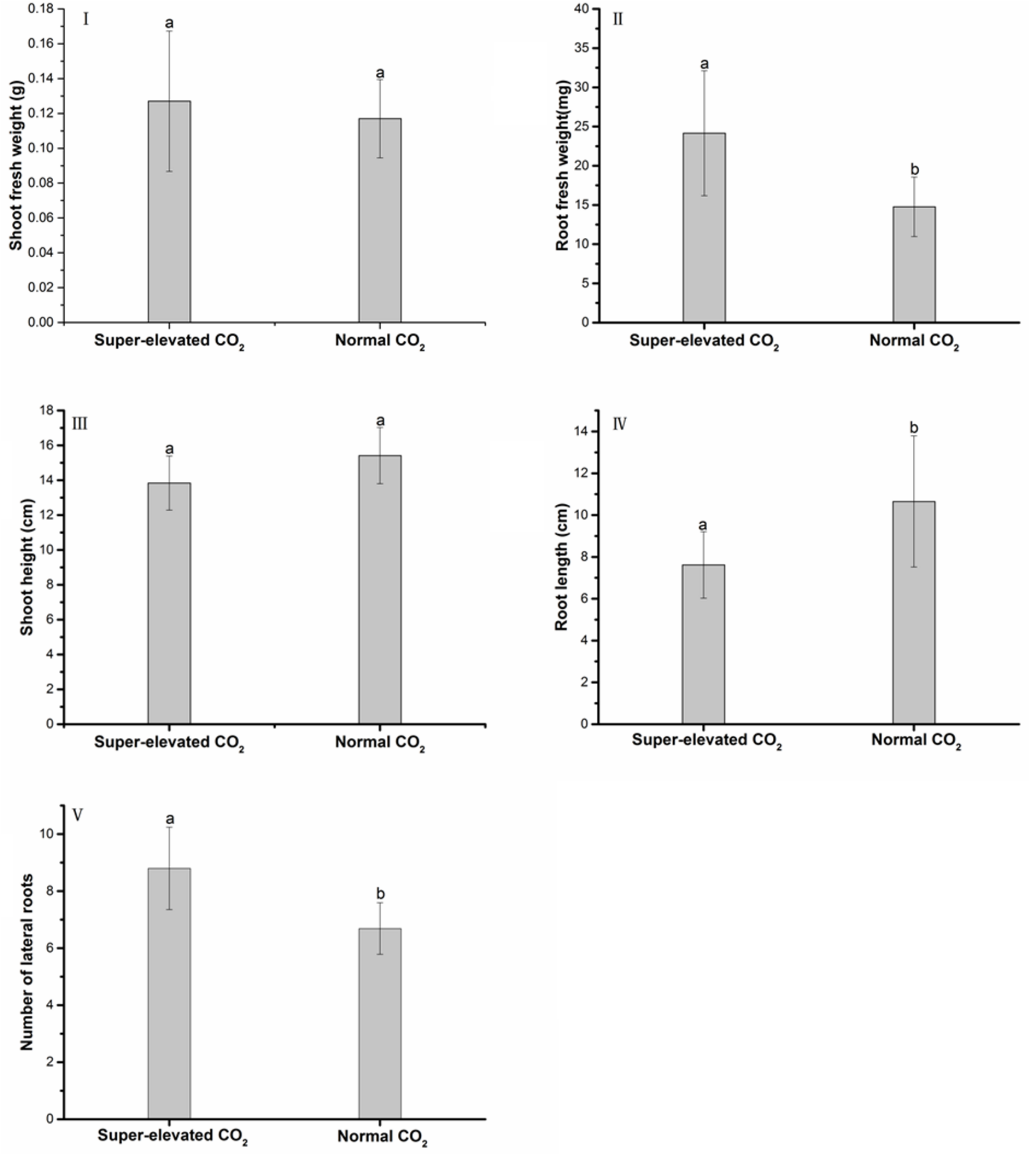
Physiological responses of wheat seedlings upon exposure to different levels of CO_2_. Values are presented as mean±SD, error bars represent standard deviation (sample size, n = 64 under super-elevated CO_2_ condition and n = 48 under normal CO_2_ condition). Lower letters represent significant difference at p<0.05 between super-elevated and normal CO_2_ treatments.

In order to further reveal the effects of different levels of CO_2_ on wheat growth, we set up the individual treatments without TiO_2_ NPs additions. As shown in Fig 4, different concentrations of CO_2_ did not change shoot fresh biomass (Fig 4I) and shoot height (Fig 4III). Root fresh weight (Fig 4II) and number of lateral roots (Fig 4V) was significantly higher in super-elevated CO_2_ than in normal CO_2_. However, The root length treated with super concentration of CO_2_ was 1.5 times as long as the ones treated with normal level of CO_2_ (Fig 4VI).

### Effects of TiO_2_ NPs on phytohormone contents

**Fig 5:**
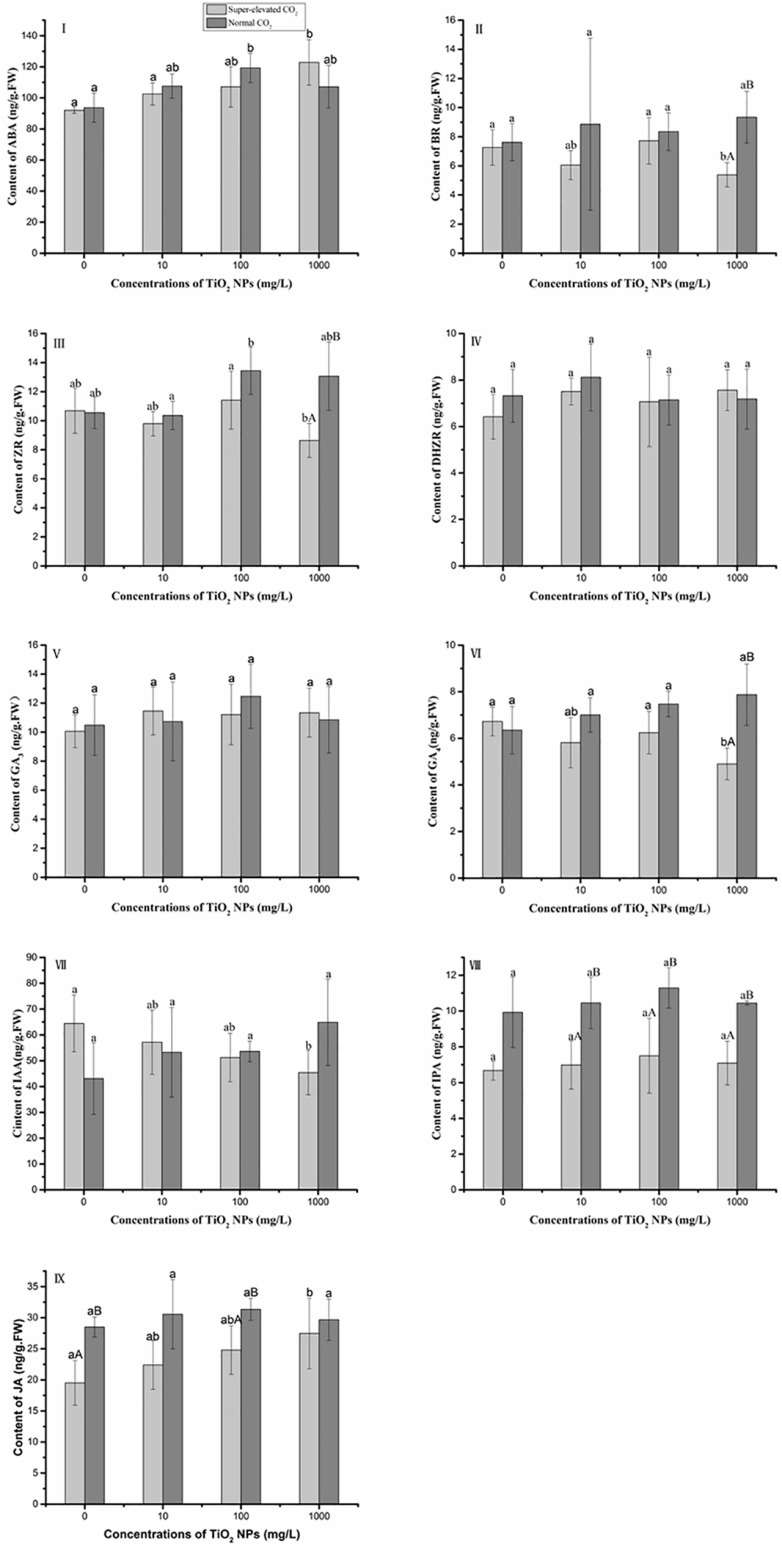
Effects of TiO_2_ NPs on phytohormone contents in wheat seedlings grown under elevated-and normal CO_2_ conditions. Data are mean±SD, error bars represent standard deviation (sample size, n = 16 for treatments under super-elevated CO_2_ condition and n = 12 for treatments under normal CO_2_ condition). Lower letters represent significant difference at p<0.05 among TiO_2_ NPs treatments under the same CO_2_ conditions; Upper letters represent significant difference at p<0.05 between elevated CO_2_ and normal CO_2_ conditions at the same TiO_2_ NPs concentration.

Phytohormones are of importance in plant growth and development [38]. The contents of different phytohormones are determined using ELISA methods in Fig 5. The ABA contents in seedlings exposed to elevated CO_2_ increased with TiO_2_ NPs concentrations increasing (Fig 5I). For plants grown under normal-CO_2_ condition, the highest ABA content (119.4±9.41 ng/g FW) was in the 100 mg/L TiO_2_ NPs treatment. Within each of the TiO_2_ NPs treatments, there was no significant difference in ABA content between the treatments with elevated level of CO_2_ and normal level of CO_2_.

BR is responsible for stem elongation and cell division in plants [39,40]. The BR contents were not changed upon exposure to the concentrations of 10 and 100 mg/L TiO_2_ NPs, regardless of the levels of CO_2_ (Fig 3II). However, when exposing to 1000 mg/L TiO_2_ NPs, the BR content in wheat seedlings treated with super-elevated CO_2_ was 42% lower than the one treated with normal CO_2_.

ZR and DHZR are two cytokines, which can regulate cell growth and inhibit senescence [38,41]. As shown in Fig 5III, the normal level of CO_2_ significantly increased the ZR contents in wheat seedlings treated with 1000 mg/L TiO_2_ NPs by 50% relative to the elevated level of CO_2_ treatment. However, neither the CO_2_ concentrations nor the TiO_2_ NPs concentrations affected the DHZR contents (Fig 3IV).

Gibberellins is another important phytohormone that can regulate cell elongation and plant growth [42]. To date, more than 100 different types of GA have been identified, although only a few such as GA_3_ and GA_4_ are bioactive. In this study, the GA_3_ content in seedlings neither differed between elevated and normal CO_2_ conditions nor among all three treatments with different concentrations of TiO_2_ NPs (Fig 5V). However, in the 1000 mg/L TiO_2_ NPs treatment, the GA_4_ content in normal CO_2_ treatment was approximately 1.6-fold of the one treated super-elevated CO_2_ (Fig 3VI).

IAA, one of the earliest discovered phytohormones, involves many physiological and biochemical processes, including cell elongation, growth, and division, and vascular tissue differentiation [43]. Under the condition of elevated CO_2_, the IAA contents decreased with TiO_2_ NPs concentrations increasing (Fig 5VII). However, the normal level of CO_2_ did not significantly alter the IAA contents among TiO_2_ NPs treatments. Another auxin, IPA, is important in stimulating root growth [44]. Similar to IAA, the super-elevated concentration of CO_2_ significantly decreased the IPA contents than the normal concentration of CO_2_, regardless of TiO_2_ NPs concentrations (Fig 5VIII).

Jasmonic acid (JA) is a lipid-derived signaling molecular that is important for plant development and responds to biotic and abiotic stresses [45,46]. The results show that under the conditions of super-elevated CO_2_, the JA contents exhibited a dose-response manner with TiO_2_ NPs concentrations increasing (Fig 5IX). However, under normal CO_2_ conditions, different concentrations of TiO_2_ NPs did not significantly change the JA contents. The JA content was much lower in seedlings treated with elevated CO_2_ than the ones with normal CO_2_, regardless of NPs concentrations.

**Fig 6:**
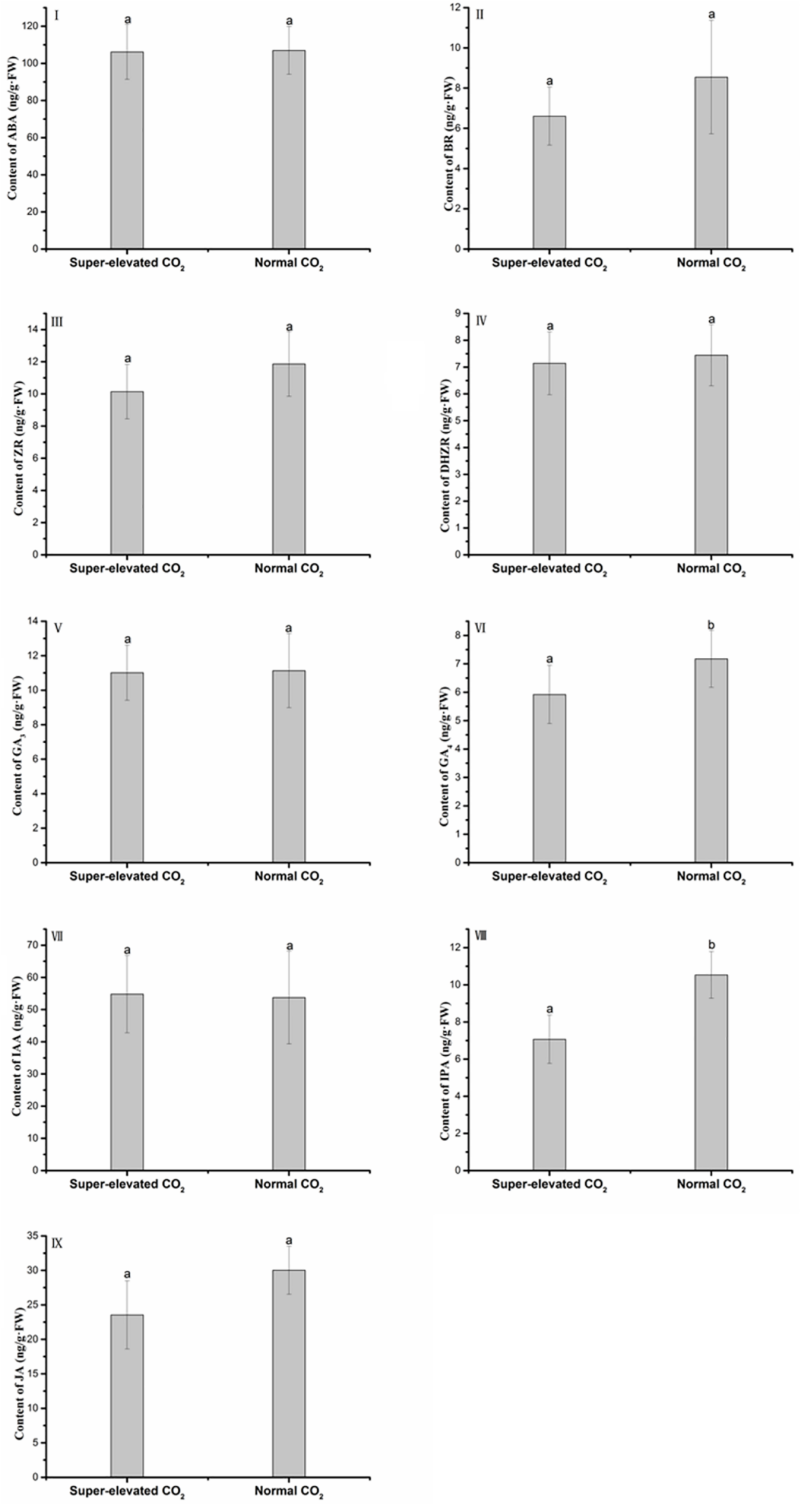
Phytohormone contents of wheat seedlings exposure to different levels of CO_2_. Values are presented as mean±SD, error bars represent standard deviation (sample size, n = 64 under super-elevated CO_2_ condition and n = 48 under normal CO_2_ condition). Lower letters represent significant difference at p<0.05 between super-elevated and normal CO_2_ treatments.

Also, as shown in Fig 6, we analysed effects of CO_2_ on phytohormone contents. Most of the hormones showed no significant difference under different CO_2_ concentrations. However, compared to normal CO_2_, the contents of GA_4_ (Fig 6VI) and IPA(Fig 6VIII) decreased under super-elevated CO_2_.

### Ti Content in wheat shoots and roots

**Fig 7:**
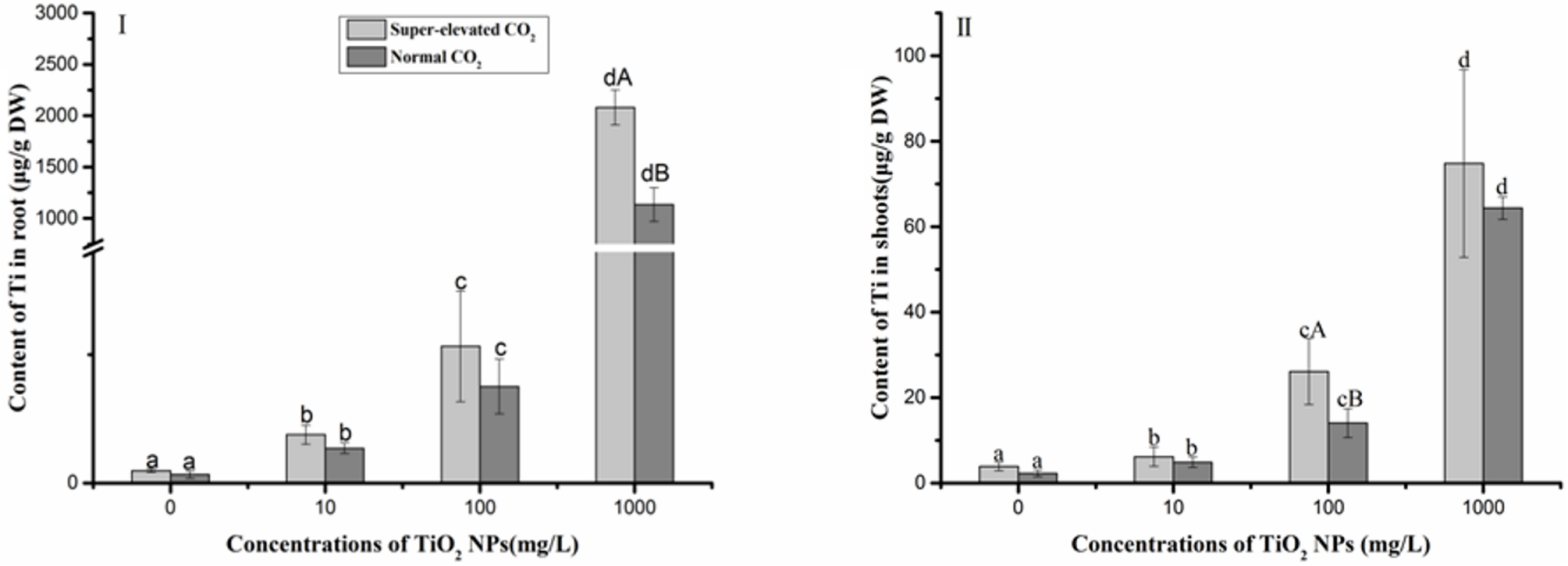
Ti contents in wheat roots and shoots. Data are mean±SD, error bars represent standard deviation (sample size, n = 3 in all treatments). Lower letters represent significant difference at p<0.05 among TiO_2_ NPs treatments under the same CO_2_ conditions; Upper letters represent significant difference at p<0.05 between super-elevated CO_2_ and normal CO_2_ conditions at the same TiO_2_ NPs concentration.

As shown in Fig 7I, a dose-response fashion of Ti accumulation in wheat roots was evident under both super-elevated and normal CO_2_ conditions. As TiO_2_ NPs concentration increased to 1000 mg/L, the Ti content in the normal CO_2_ treatment was significantly lower than in the super-elevated CO_2_ treatment, implying that the excess amounts of CO_2_ promoted Ti uptake. The pattern of Ti distribution in wheat shoots was similar to the roots (Fig 7II). At the 100 mg/L TiO_2_ NPs, the elevated level of CO_2_ resulted in more Ti translocation to shoots from roots as compared to the normal level of CO_2_.

## Conclusions

When seedlings were exposed to NPs, most of NPs aggregated on the surface of roots [47,48], which led to reduction of hydraulic conductivity and water availability to plants, and subsequently lower transpiration rate and inhibit plant development. Compared to the normal CO_2_ conditions, wheat seedlings treated with the elevated level of CO_2_ exhibited higher root biomass and formed more lateral roots. Under both elevated and normal CO_2_ conditions, the ABA content increased with the concentrations of TiO_2_ NPs increasing, but the CO_2_ levels did not alter the ABA content in NPs treated wheat seedlings. The combined effects of elevated CO_2_ and high TiO_2_ NPs concentrations caused decreases of BR, ZR, and GA_3_ contents, while neither CO_2_ nor TiO_2_ NPs negatively affected the phytohormone level. The IPA and JA contents were lower in plants grown under super-elevated CO_2_ conditions, and the JA content increased with increasing TiO_2_ NPs concentrations. The Ti contents showed a dose-response manner in both shoots and roots, and the levels of CO_2_ could alter Ti accumulation and distribution at certain exposure concentrations of TiO_2_ NPs. The study is helpful in understanding effects of TiO_2_ NPs on plants under different CO_2_ conditions.

## Supporting information

S1 Fig TEM images of TiO_2_ NPs. (TIF)

S1 Table Shoot fresh biomass. Values are mean ± SD (n≥3). Letters represent significant difference (p<0.05) among TiO_2_ NPs treatments under the same growth conditions; * represents significant difference (p<0.05) between super-elevated CO_2_ and normal CO_2_ conditions at each TiO_2_ NPs concentration.
(PDF)

S2 Table Root fresh biomass. Values are mean ± SD (n≥3). Letters represent significant difference (p<0.05) among TiO_2_ NPs treatments under the same growth conditions; * represents significant difference (p<0.05) between super-elevated CO_2_ and normal CO_2_ conditions at each TiO_2_ NPs concentration.
(PDF)

S3 Table Number of lateral roots. Values are mean ± SD (n≥3). Letters represent significant difference (p<0.05) among TiO_2_ NPs treatments under the same growth conditions; * represents significant difference (p<0.05) between super-elevated CO_2_ and normal CO_2_ conditions at each TiO_2_ NPs concentration.
(PDF)

S4 Table Shoot height. Values are mean ± SD (n≥3). Letters represent significant difference (p<0.05) among TiO_2_ NPs treatments under the same growth conditions; * represents significant difference (p<0.05) between super-elevated CO_2_ and normal CO_2_ conditions at each TiO_2_ NPs concentration.
(PDF)

S5 Table Root length. Values are mean ± SD (n≥3). Letters represent significant difference (p<0.05) among TiO_2_ NPs treatments under the same growth conditions; * represents significant difference (p<0.05) between super-elevated CO_2_ and normal CO_2_ conditions at each TiO_2_ NPs concentration.
(PDF)

S6 Table Effects of TiO_2_ NPs on phytohormone contents. Values are mean ± SD (n = 3).Lowercase letters represent significant difference (p<0.05) among TiO_2_ NPs treatments under the same growth conditions; Uppercase letters represent significant difference (p<0.05) between super-elevated CO_2_ and normal CO_2_ conditions at each TiO_2_ NPs concentration.
(PDF)

S7 Table Content of Ti in shoots. Content of Ti in roots. Values are mean ± SD (n≥3). Letters represent significant difference (p<0.05) among TiO_2_ NPs treatments under the same growth conditions; * represents significant difference (p<0.05) between super-elevated CO_2_ and normal CO_2_ conditions at each TiO_2_ NPs concentration.
(PDF)

S8 Table Content of Ti in roots. Values are mean ± SD (n≥3). Letters represent significant difference (p<0.05) among TiO_2_ NPs treatments under the same growth conditions; * represents significant difference (p<0.05) between super-elevated CO_2_ and normal CO_2_ conditions at each TiO_2_ NPs concentration.
(PDF)

S1 Data The data for all of the experiments. (PDF)
