# Supplementary material for: Effects of TiO2 nanoparticles on wheat (Triticum aestivum L.) seedlings cultivated under super-elevated and normal CO2 conditions
Source: PLoS One. 2017 May 30;12(5):e0178088. doi: 10.1371/journal.pone.0178088 (PMC5448767; doi:10.1371/journal.pone.0178088)
Supplement: S1 Table — Values are mean ± SD (n≥3). Letters represent significant difference (p<0.05) among TiO2 NPs treatments under the same growth conditions; * represents significant difference (p<0.05) between super-elevated CO2 and normal CO2 conditions at each TiO2 NPs concentration. (PDF) [file pone.0178088.s002.pdf]

S1 Table. Shoot fresh biomass

NPs Concentration (mg/L)	CK		10		100		1000	
	Mean±SD	95% Confidence Interval for Mean	Mean±SD	95% Confidence Interval for Mean	Mean±SD	95% Confidence Interval for Mean	Mean±SD	95% Confidence Interval for Mean
Super-elevated CO_2/g	0.1504±0.0467a	0.1208-0.1801	0.1426±0.0281a	0.1248-0.1604	0.1077±0.0391b	0.0829-0.1326	0.1072±0.0272b	0.0900-0.1245
Normal CO_2/g	0.1137±0.0255a*	0.0941-0.1333	0.1270±0.0292a	0.1046-0.1495	0.1145±0.0164a	0.1019-0.1272	0.1127±0.0167a	0.0998-0.1256

Values are mean ± SD (n⩾3). Letters represent significant difference ($p<0.05$) among TiO_2 NPs treatments under the same growth conditions; * represents significant difference ($p<0.05$) between super-elevated CO_2 and normal CO_2 conditions at each TiO_2 NPs concentration
