# Supplementary material for: Effects of TiO2 nanoparticles on wheat (Triticum aestivum L.) seedlings cultivated under super-elevated and normal CO2 conditions
Source: PLoS One. 2017 May 30;12(5):e0178088. doi: 10.1371/journal.pone.0178088 (PMC5448767; doi:10.1371/journal.pone.0178088)
Supplement: S2 Table — Values are mean ± SD (n≥3). Letters represent significant difference (p<0.05) among TiO2 NPs treatments under the same growth conditions; * represents significant difference (p<0.05) between super-elevated CO2 and normal CO2 conditions at each TiO2 NPs concentration. (PDF) [file pone.0178088.s003.pdf]

S2 Table. Root fresh biomass

NPs Concentration (mg/L)	CK		10		100		1000	
	Mean±SD	95% Confidence Interval for Mean	Mean±SD	95% Confidence Interval for Mean	Mean±SD	95% Confidence Interval for Mean	Mean±SD	95% Confidence Interval for Mean
Super-elevated CO_2/g	0.0244±0.0042a	0.02003—0.02822	0.0261±0.0051a	0.0212-0.0311	0.0208±0.0051a	0.0163-0.0254	0.0232±0.0048a	0.0187-0.0276
Normal CO_2/g	0.0161±0.0027a*	0.0140-0.0182	0.0179±0.0036a*	0.0149-0.0209	0.0128±0.0026b	0.0107-0.0150	0.0126±0.0015b*	0.0113-0.0139

Values are mean ± SD (n⩾3). Letters represent significant difference ($p<0.05$) among TiO_2 NPs treatments under the same growth conditions; * represents significant difference ($p<0.05$) between super-elevated CO_2 and normal CO_2 conditions at each TiO_2 NPs concentration.
