# Supplementary material for: Effects of TiO2 nanoparticles on wheat (Triticum aestivum L.) seedlings cultivated under super-elevated and normal CO2 conditions
Source: PLoS One. 2017 May 30;12(5):e0178088. doi: 10.1371/journal.pone.0178088 (PMC5448767; doi:10.1371/journal.pone.0178088)
Supplement: S3 Table — Values are mean ± SD (n≥3). Letters represent significant difference (p<0.05) among TiO2 NPs treatments under the same growth conditions; * represents significant difference (p<0.05) between super-elevated CO2 and normal CO2 conditions at each TiO2 NPs concentration. (PDF) [file pone.0178088.s004.pdf]

S3 Table. Number of lateral roots

NPs Concentration (mg/L)	CK		10		100		1000	
	Mean±SD	95% Confidence Interval for Mean	Mean±SD	95% Confidence Interval for Mean	Mean±SD	95% Confidence Interval for Mean	Mean±SD	95% Confidence Interval for Mean
Super-elevated CO_2	9.13±1.45a	8.35-9.90	8.63±1.5a	7.80-9.45	8.94±1.24a	8.28-9.60	8.50±1.26a	7.83-9.17
Normal CO_2	7.08±1.08a*	6.39-7.77	6.67±0.65 a*	6.25-7.08	6.67±0.98 a*	6.04-7.29	6.33±0.78 a*	5.84-6.83

Values are mean ± SD (n⩾3). Letters represent significant difference ($p<0.05$) among TiO_2 NPs treatments under the same growth conditions; * represents significant difference ($p<0.05$) between super-elevated CO_2 and normal CO_2 conditions at each TiO_2 NPs concentration.
