# Supplementary material for: Effects of TiO2 nanoparticles on wheat (Triticum aestivum L.) seedlings cultivated under super-elevated and normal CO2 conditions
Source: PLoS One. 2017 May 30;12(5):e0178088. doi: 10.1371/journal.pone.0178088 (PMC5448767; doi:10.1371/journal.pone.0178088)
Supplement: S4 Table — Values are mean ± SD (n≥3). Letters represent significant difference (p<0.05) among TiO2 NPs treatments under the same growth conditions; * represents significant difference (p<0.05) between super-elevated CO2 and normal CO2 conditions at each TiO2 NPs concentration. (PDF) [file pone.0178088.s005.pdf]

S4 Table. Shoot height

NPs Concentration (mg/L)	CK		10		100		1000	
	Mean±SD	95% Confidence Interval for Mean	Mean±SD	95% Confidence Interval for Mean	Mean±SD	95% Confidence Interval for Mean	Mean±SD	95% Confidence Interval for Mean
Super-elevated CO_2/cm	14.34±1.78a	13.39-15.28	13.77±1.30a	13.08-14.46	14.10±1.67a	13.21-14.99	13.16± 1.30a	12.46-13.85
Normal CO_2/cm	15.41±1.60	14.39-16.42	15.50±1.90	14.29-16.71	15.48±1.56	14.49-16.48	15.27±1.60	14.25-16.28

Values are mean ± SD (n⩾3). Letters represent significant difference ($p<0.05$) among TiO_2 NPs treatments under the same growth conditions; * represents significant difference ($p<0.05$) between super-elevated CO_2 and normal CO_2 conditions at each TiO_2 NPs concentration.
