# Supplementary material for: Effects of TiO2 nanoparticles on wheat (Triticum aestivum L.) seedlings cultivated under super-elevated and normal CO2 conditions
Source: PLoS One. 2017 May 30;12(5):e0178088. doi: 10.1371/journal.pone.0178088 (PMC5448767; doi:10.1371/journal.pone.0178088)
Supplement: S6 Table — Values are mean ± SD (n = 3).Lowercase letters represent significant difference (p<0.05) among TiO2 NPs treatments under the same growth conditions; Uppercase letters represent significant difference (p<0.05) between super-elevated CO2 and normal CO2 conditions at each TiO2 NPs concentration. (PDF) [file pone.0178088.s007.pdf]

S6 Table. Effects of TiO_2 NPs on phytohormone contents

		ABA (ng/g.FW)	BR (ng/g.FW)	ZR (ng/g.FW)	DHZR (ng/g.FW)	GA_3 (ng/g.FW)	GA_4 (ng/g.FW)	IAA (ng/g.FW)	IPA (ng/g.FW)	JA (ng/g.FW)
Super-elevated CO_2 (mg/L)	CK	92.01±2.05a	7.26±1.22a	10.69±0.55ab	6.42±0.96a	10.05±1.12a	6.72±0.61a	64.43±11.00a	6.67±0.53a	19.51±3.60aA
	10	102.53±7.20a	6.05±0.99ab	9.79±0.84ab	7.51±0.58a	11.44±1.64a	5.81±1.08ab	57.18±12.44ab	6.99±1.35aA	22.40±3.92ab
	100	107.13±13.01ab	7.72±1.59a	11.42±2.00a	7.07±1.93a	11.21±2.08a	6.24±0.91a	51.20±9.40ab	7.50±2.10aA	24.78±3.90abA
	1000	122.82±14.57b	5.38±0.83bA	8.64±1.17bA	7.57±0.89a	11.33±1.68a	4.90±0.68bA	45.42±8.61b	7.09±1.22aA	27.46±5.68b
Normal CO_2 (mg/L)	CK	93.67±9.29a	7.63±1.28a	10.55±1.09ab	7.32±1.14a	10.48±2.08a	6.35±1.02a	43.11±13.85a	9.93±1.97a	28.50±1.62aB
	10	107.61±7.78ab	8.86±5.90a	10.37±0.98a	8.12±1.44a	10.73±2.71a	7.00±0.74a	53.27±17.36a	10.45±1.43aB	30.56±5.55a
	100	119.29±9.41b	8.35±1.29a	13.44±1.63b	7.15±1.08a	12.46±2.21a	7.47±0.55a	53.63±4.01a	11.29±1.13aB	31.34±1.77aB
	1000	107.20±13.65ab	9.34±1.77aB	13.07±2.35abB	7.18±1.29a	10.85±2.29a	7.87±1.32aB	64.88±16.69a	10.45±0.11aB	29.67±3.32a

Values are mean ± SD (n=3).Lowercase letters represent significant difference ($p<0.05$) among TiO_2 NPs treatments under the same growth conditions; Uppercase letters represent significant difference ($p<0.05$) between super-elevated CO_2 and normal CO_2 conditions at each TiO_2 NPs concentration.
