# Supplementary material for: Effects of TiO2 nanoparticles on wheat (Triticum aestivum L.) seedlings cultivated under super-elevated and normal CO2 conditions
Source: PLoS One. 2017 May 30;12(5):e0178088. doi: 10.1371/journal.pone.0178088 (PMC5448767; doi:10.1371/journal.pone.0178088)
Supplement: S7 Table — Content of Ti in roots. Values are mean ± SD (n≥3). Letters represent significant difference (p<0.05) among TiO2 NPs treatments under the same growth conditions; * represents significant difference (p<0.05) between super-elevated CO2 and normal CO2 conditions at each TiO2 NPs concentration. (PDF) [file pone.0178088.s008.pdf]

S7 Table. Content of Ti in shoots

NPs Concentration (mg/L)	CK		10		100		1000	
	Mean±SD	95% Confidence Interval for Mean	Mean±SD	95% Confidence Interval for Mean	Mean±SD	95% Confidence Interval for Mean	Mean±SD	95% Confidence Interval for Mean
Super-elevated CO_2/cm	3.89±1.00a	1.32- 6.47	6.17±2.26b	-14.16-26.50	26.11±7.75c	6.87-45.36	74.79±21.92d	20.33-129.25
Normal CO_2/cm	2.28±0.81a	0.26-4.29	4.89±1.22b	1.87-7.91	14.06±3.36c*	-16.12-44.23	64.36±2.60d	57.90-70.82

Values are mean ± SD (n⩾3). Letters represent significant difference ($p<0.05$) among TiO_2 NPs treatments under the same growth conditions; * represents significant difference ($p<0.05$) between super-elevated CO_2 and normal CO_2 conditions at each TiO_2 NPs concentration.
