# Supplementary material for: Effects of TiO2 nanoparticles on wheat (Triticum aestivum L.) seedlings cultivated under super-elevated and normal CO2 conditions
Source: PLoS One. 2017 May 30;12(5):e0178088. doi: 10.1371/journal.pone.0178088 (PMC5448767; doi:10.1371/journal.pone.0178088)
Supplement: S8 Table — Values are mean ± SD (n≥3). Letters represent significant difference (p<0.05) among TiO2 NPs treatments under the same growth conditions; * represents significant difference (p<0.05) between super-elevated CO2 and normal CO2 conditions at each TiO2 NPs concentration. (PDF) [file pone.0178088.s009.pdf]

S8 Table. Content of Ti in roots

NPs Concentration (mg/L)	CK		10		100		1000	
	Mean±SD	95% Confidence Interval for Mean	Mean±SD	95% Confidence Interval for Mean	Mean±SD	95% Confidence Interval for Mean	Mean±SD	95% Confidence Interval for Mean
Super-elevated CO_2/cm	24.26±3.24a	-4.84-53.36	94.30±18.73b	47.78-140.83	266.64±107.66c	-0.800-534.09	2080.98±170.79d	546.51-3615.44
Normal CO_2/cm	16.60±6.99a	-46.17-79.37	67.80±9.98b	-21.91-157.51	188.27±53.30c	55.87-320.66	1134.49±165.46d*	-352.14-2621.12

Values are mean ± SD (n⩾3). Letters represent significant difference ($p<0.05$) among TiO_2 NPs treatments under the same growth conditions; * represents significant difference ($p<0.05$) between super-elevated CO_2 and normal CO_2 (plant growth chamber) conditions at each TiO_2 NPs concentration.
