# Supplementary material for: Effects of TiO2 nanoparticles on wheat (Triticum aestivum L.) seedlings cultivated under super-elevated and normal CO2 conditions
Source: PLoS One. 2017 May 30;12(5):e0178088. doi: 10.1371/journal.pone.0178088 (PMC5448767; doi:10.1371/journal.pone.0178088)
Supplement: S1 Data — (PDF) [file pone.0178088.s010.pdf]

	Treatment	Content of ABA(ng/g.FW)	Content of IAA(ng/g.FW)	Content of ZR(ng/g.FW)	Content of GA3(ng/g.FW)	Content of BR(ng/g.FW)	Content of JA(ng/g.FW)	Content of IPA(ng/g.FW)	Content of GA4(ng/g.FW)	Content of DNZR(ng/g.FW)
Super-elevated CO_2	CK-1	91.04	68.57	9.44	9.72	5.91	18.90	6.05	6.03	6.74
	CK-2	89.66	59.43	12.11	10.37	7.06	16.81	6.54	6.38	5.90
	CK-3	93.14	52.21	9.25	11.39	7.20	17.56	6.76	7.21	5.43
	CK-4	94.21	77.52	11.96	8.74	8.87	24.74	7.34	7.26	7.61
	10-1	92.44	60.11	8.70	11.39	5.54	18.90	6.74	6.22	7.63
	10-2	103.81	47.46	9.66	10.94	4.91	26.12	7.08	4.41	6.94
	10-3	104.40	47.57	10.11	13.68	6.66	19.11	5.42	5.66	7.21
	10-4	109.48	73.57	10.70	9.76	7.07	25.45	8.71	6.97	8.26
	100-1	106.40	47.35	10.39	12.96	7.06	26.93	5.67	5.34	6.41
	100-2	103.61	61.91	12.06	10.22	8.37	25.40	9.31	6.66	5.95
	100-3	93.67	44.33	9.32	12.91	5.89	19.11	5.69	5.65	5.96
	100-4	124.84	98.40	13.90	8.75	9.57	27.70	9.33	7.32	9.94
	1000-1	143.16	48.99	9.72	13.30	5.38	21.96	6.88	4.27	7.46
	1000-2	118.36	36.80	8.56	10.39	4.75	35.40	8.76	4.82	7.08
	1000-3	108.65	40.12	7.03	12.08	4.84	25.79	5.81	4.64	6.88
	1000	121.10	55.76	9.25	9.56	6.56	26.70	6.93	5.86	8.84

	-4									
Normal CO_2	CK-1	88.14	36.47	11.02	12.88	6.24	27.88	8.58	5.97	8.13
	CK-2	104.40	59.02	11.32	9.21	8.78	30.34	12.20	7.50	7.82
	CK-3	88.48	33.83	9.30	9.35	7.86	27.28	9.01	5.58	6.01
	10-1	101.46	69.99	11.37	8.63	15.58	31.35	11.29	7.85	9.76
	10-2	105.00	35.32	9.42	13.79	4.50	23.59	8.80	6.50	7.09
	10-3	116.35	54.51	10.31	9.78	6.50	36.73	11.27	6.66	7.50
	100-1	126.51	57.05	13.44	13.30	7.53	33.17	11.92	8.03	6.40
	100-2	108.65	54.64	11.81	9.96	7.68	29.63	11.98	7.44	8.39
	100-3	122.72	49.22	15.08	14.13	9.84	31.21	9.99	6.94	6.66
	1000-1	108.45	47.35	11.76	10.77	7.43	30.34	10.58	6.95	5.72
	1000-2	92.96	80.57	15.78	13.17	10.94	26.07	10.37	9.39	8.15
	1000-3	120.18	66.73	11.66	8.60	9.65	32.60	10.39	7.27	7.68

	Treatment	Shoot Ti content (μg/g DW)	Root Ti content
Super-elevated CO_2	CK-1	4.08	27.98
	CK-2	3.86	22.08
	CK-3	3.72	22.71
	10-1	8.34	102.39
	10-2	3.83	107.63
	10-3	6.35	72.89
	100-1	21.65	385.00
	100-2	27.63	240.40
	100-3	29.06	174.53
	1000-1	58.84	2201.74
	1000-2	99.79	1960.21
	1000-3	65.74	2596.05
Normal CO_2	CK-1	3.21	11.66
	CK-2	1.75	17.00
	CK-3	1.87	21.00
	10-1	3.55	60.87
	10-2	5.20	63.21
	10-3	5.92	79.20
	100-1	11.68	127.16
	100-2	16.43	225.14
	100-3	14.05	212.50
	1000-1	61.66	1017.49
	1000-2	64.56	1251.49
	1000-3	66.85	1100.00

		number of lateral root	Shoot biomass(g)	root biomass	Shoot height(cm)	Root length
Super-elevated CO2	CK-1	9	0.1364	0.0191	12	5.2
	CK-2	8	0.17	0.0288	14.6	8.1
	CK-3	10	0.1417	0.037	15.5	8.6
	CK-4	10	0.2088	0.0303	12.8	5.1
	CK-5	9	0.2055	0.0383	16.6	10.6
	CK-6	7	0.2146	0.0198	17.5	7.4
	CK-7	10	0.1566	0.0211	16.3	8.6
	CK-8	9	0.0958	0.0152	16.1	10.5
	CK-9	11	0.0795	0.0236	11.3	6.1
	CK-10	8	0.1503	0.0262	13	10.3
	CK-11	12	0.0832	0.026	15.5	5.6
	CK-12	10	0.1629	\	13	5.2
	CK-13	10	\	\	13.6	8.1
	CK-14	7	\	\	13.8	5.2
	CK-15	7	\	\	13.3	5.2
	CK-16	\	\	\	14.5	6.7
	10-1	9	0.1732	0.0293	15.1	10.9
	10-2	9	0.1619	0.0184	15.3	7.6
	10-3	11	0.1681	0.0186	15.1	8.9
	10-4	8	0.138	0.0275	12.1	8.7
	10-5	6	0.0955	0.0177	15.5	6.5
	10-6	8	0.1711	0.0305	14.5	8.4
	10-7	8	0.1084	0.018	12.8	7.4
	10-8	9	0.1621	0.0277	14.2	8.9

	10-9	9	0.1363	0.0309	12.5	8.9
	10-10	10	0.163	0.0365	13.2	4.5
	10-11	6	0.1043	\	11.3	7.1
	10-12	12	0.1295	\	13.4	8.2
	10-13	8	\	\	13.4	9.5
	10-14	9	\	\	13	9
	10-15	8	\	\	15.5	9.6
	10-16	8	\	\	13.4	12
	100-1	8	0.0555	0.0178	13.9	6.4
	100-2	7	0.0834	0.0318	15.5	8.6
	100-3	10	0.1487	0.0197	13.8	8.3
	100-4	8	0.1091	0.0242	13.1	6
	100-5	10	0.1176	0.0172	16.4	8.8
	100-6	9	0.1388	0.0235	16.9	8.2
	100-7	8	0.0777	0.016	14.5	8.7
	100-8	8	0.1885	0.0165	12.1	8
	100-9	11	0.0644	0.0003	14.1	9.2
	100-10	11	0.0753	0.0206	12.9	7.3
	100-11	9	0.1277	\	11	6.7
	100-12	9	0.1058	\	14.2	8.5
	100-13	9	\	\	16.3	5.9
	100-14	9	\	\	13.6	7.5
	100-15	7	\	\	12.1	6.1
	100-16	10	\	\	15.2	7
	1000-1	10	0.1085	0.0185	13.5	7.5

	1000-2	8	0.0545	0.0257	10.2	6.2
	1000-3	9	0.1449	0.0358	13.2	5.2
	1000-4	10	0.1412	0.0365	13.5	5.9
	1000-5	9	0.1221	0.038	15.6	8
	1000-6	10	0.12	0.0204	13.5	8.1
	1000-7	10	0.1077	0.0187	15	8.1
	1000-8	9	0.0883	0.0139	12.1	7.6
	1000-9	9	0.0738	0.0255	13.4	6.6
	1000-10	6	0.0911	0.0214	12.4	6
	1000-11	7	0.1025	0.0319	14.2	7
	1000-12	8	0.1323	\	13.6	7.7
	100-13	7	\	\	12.8	7
	100-14	7	\	\	13.1	6.2
	100-15	9	\	\	11.2	7.8
	100-16	8	\	\	13.2	8.9
Normal CO_2	CK-1	8	0.1285	0.016	14.5	15.8
	CK-2	7	0.0816	0.015	19	17
	CK-3	6	0.0973	0.015	17.3	18.1
	CK-4	6	0.0987	0.0144	15.5	12.1
	CK-5	6	0.0874	0.0126	13.7	7.8
	CK-6	6	0.1099	0.0136	14.2	7
	CK-7	8	0.125	0.0203	15.5	6.1
	CK-8	7	0.1609	0.0188	14.8	7
	CK-9	9	0.1337	0.0192	14.8	11
	CK-10	8	\	\	17.1	10.2

	CK-11	8	\	\	14.1	15.8
	CK-12	6	\	\	14.4	11.9
	10-1	6	0.1594	0.0237	16.2	13
	10-2	7	0.159	0.0211	14.8	9.5
	10-3	7	0.124	0.0128	19	10.8
	10-4	6	0.1543	0.0143	16	10.2
	10-5	8	0.1256	0.0156	17.4	9.1
	10-6	7	0.072	0.0198	14	10.1
	10-7	7	0.1191	0.0176	17.2	8.8
	10-8	6	0.0975	0.0184	14.1	7.5
	10-9	6	0.1325	\	11.8	12.8
	10-10	6	\	\	14.5	12.2
	10-11	7	\	\	16.1	12.5
	10-12	7	\	\	14.9	9.9
	100-1	8	0.1237	0.0146	19.3	9.1
	100-2	7	0.1175	0.0151	16.4	8.7
	100-3	8	0.1097	0.0088	15.3	8.5
	100-4	6	0.1097	0.0148	16	8.8
	100-5	6	0.1146	0.0153	15.3	11.5
	100-6	5	0.083	0.0121	13.1	13.1
	100-7	6	0.1401	0.0122	15.2	16.1
	100-8	6	0.1297	0.0096	15	17.6
	100-9	8	0.1028	\	15.5	8.1
	100-10	6	\	\	13.3	8.3
	100-11	7	\	\	15.3	8.8

	100-12	7	\	\	16.1	8.7
	1000-1	7	0.1469	0.0133	16.5	14.5
	1000-2	7	0.1166	0.0144	15.5	14.1
	1000-3	7	0.1009	0.0107	17.3	9.6
	1000-4	7	0.1066	0.0133	15.4	14.5
	1000-5	6	0.1286	0.0119	16.9	7.7
	1000-6	5	0.1001	0.0109	14.3	9.8
	1000-7	7	0.1138	0.0135	14.5	8.2
	1000-8	6	0.0913	\	12.7	7.8
	1000-9	6	0.1094	\	15.5	7
	1000-10	5	\	\	14.9	7.6
	1000-11	7	\	\	17.2	9.7
	1000-12	6	\	\	12.5	7.3
